# Supplementary material for: FastqPuri: high-performance preprocessing of RNA-seq data
Source: BMC Bioinformatics. 2019 May 3;20:226. doi: 10.1186/s12859-019-2799-0 (PMC6500068; doi:10.1186/s12859-019-2799-0)
Supplement: Supplementary file 2 — Archive of FastqPuri. Archive containing all files needed to install and run FastqPuri v1.0.6. Date stamp March 22, 2019. (GZ 47,819 kb) [file 12859_2019_2799_MOESM2_ESM.gz › FastqPuri-1.0.6/html/struct__adapter.html]

FastqPuri: \_adapter Struct Reference


FastqPuri

Public Attributes |
List of all members

\_adapter Struct Reference

`#include <struct_trimFilter.h>`

|  |  |
| --- | --- |
| Public Attributes | |
| char \* | ad\_fa |
|  | |
| char \* | ad2\_fa |
|  | |
| int | mismatches |
|  | |
| double | threshold |
|  | |
| int | Nad |
|  | |

## Detailed Description

@ brief adapter struct

## Member Data Documentation

## ◆ ad2\_fa

char\* \_adapter::ad2\_fa

fasta file containing adapters from read 2

## ◆ ad\_fa

char\* \_adapter::ad\_fa

fasta file containing adapters

## ◆ mismatches

int \_adapter::mismatches

Number of allowed mismatches

## ◆ Nad

int \_adapter::Nad

Number of adapters

## ◆ threshold

double \_adapter::threshold

Score threshold

---

The documentation for this struct was generated from the following file:

- include/struct\_trimFilter.h


---

Generated on Mon Mar 19 2018 23:42:01 for FastqPuri by  

 1.8.14
